# Implementation of a Bundle of Care in Colorectal Surgery to Reduce Surgical Site Infections Successfully at Cantonal Hospital Lucerne: Study Protocol for a Prospective Observational Study

**DOI:** 10.29337/ijsp.150

**Published:** 2021-09-23

**Authors:** Stefanie Brunner, Jule Liesenberg, Lana Fourie, Jürg Metzger, Andreas Scheiwiller, Irin Zschokke, Dirk Lehnick, Jörn-Markus Gass

**Affiliations:** 1 University Hospital Cologne, General, visceral, tumor and transplant Surgery, Cologne, Germany; 2 Cantonal Hospital Lucerne, General and Visceral Surgery, Lucerne, Switzerland; 3 Department of Visceral Surgery, Clarunis University Center for Gastrointestinal and Liver Diseases, St. Clara Hospital and University Hospital Basel, Basel, Switzerland; 4 Clinical Trial Unit Central Switzerland, University of Lucerne, Lucerne, Switzerland; 5 Department of Health Sciences and Medicine, University of Lucerne, Lucerne, Switzerland

**Keywords:** colorectal surgery, bundle of care, surgical site infection

## Abstract

**Background:**

Surgical site infections (SSIs) remain a relevant problem in colorectal surgery. The aim of this study is to implement a bundle of care in order to reduce SSIs in colorectal surgery.

**Methods:**

All patients undergoing colorectal surgery between October 2018 and December 2021 will be included in a prospective observational study. Since our colorectal bundle has been established gradually, patients will be grouped in a pre-implementation (2018–2019), implementation (2019–2020) and post implementation phase (2021), in order to assess the effectiveness of the actions undertaken. Primary endpoint of this study will be surgical site infection (SSI) rate, while secondary endpoints encompass potential risk factors for SSIs. We assume that obesity, age, diabetes, alcoholism and smoking may lead to a higher risk for SSIs.

**Discussion:**

This study aims to determine whether the colorectal bundle designed and implemented at Cantonal Hospital Lucerne, will lead to a significant reduction of SSIs. The impact of potential risk factors for SSIs will additionally be evaluated.

**Trial registration:**

ClinicalTrials.gov, ID: NCT04677686. Registered retrospectively 18 December 2020.

**Highlights:**

- A bundle of care might reduce the occurence of surgical site infections after colorectal surgery.
- Analysis of risk factors may detect patient’s with high probability of developing surgical site infections.

## Background

SSIs are a common problem in the healthcare system [1]. They lead to an increased mortality and morbidity rate. Apart from the negative impact on patients, SSIs cause high healthcare costs due to prolonged hospital stay and readmission of patients. Colorectal surgery, in particular, is associated with a high infection rate ranging from 15% to 30% [2,3].

In order to understand the causes of SSIs, risk factors have to be evaluated. Intrinsic and extrinsic factors have previously been defined [4]. Intrinsic risk factors are patient-associated and can be subclassified in modifiable and non-modifiable features. Modifiable intrinsic risk factors include diabetes, alcoholism, smoking status, obesity and preoperative albumin level. Examples of non-modifiable risk factors include age and recent radiotherapy. Extrinsic risk factors are procedure-related and involve emergency surgeries or facility risk factors. There are several strategies to prevent SSIs. The biggest impact has been found in the combination of different measures into a bundle of care [5,2,6].

The Cantonal Hospital Lucerne (LUKS) is an 870-bed public acute care teaching hospital serving a population of about 500.000 people in central Switzerland. From October 2016 till September 2017 the rate of SSIs in colorectal surgery in LUKS was 18.1% whereas the average rate of SSIs in Switzerland was 13.7% during the same period of time [7]. These nationwide data were collected and analyzed by Swissnoso, an organization monitoring SSIs cross-disciplinary.

In order to reduce the SSIs at LUKS, we created a colorectal bundle containing 9 measures. These measures were based on a literature research and selected in cooperation with the Department of Hospital Infection Control.

## Methods and design

### Study design

This prospective observational monocentric study will commence at LUKS, a large non-university hospital in Switzerland. A total of 150 patients will be included between January 2021 and December 2021. Ethical approval has been obtained from the responsible Swiss Ethics Committee (EKNZ) with the project ID: 2020–00051. The study is registered at ClinicalTrials.gov with the number NCT04677686.

### Patient population

All patients undergoing colorectal surgery from January 2021 until December 2021, fulfilling the inclusion criteria and having signed an informed consent form will be included in the study. According to our annual case load, we expect to enroll about 150 patients in the study. The data of the patients which are prospectively included in this study will be compared with the data analyzed by Swissnoso (October 2017–September 2018 and October 2018–September 2019). From 2017–2018 153 patients were included and 31 infections were detected (5 superficial, 8 deep and 18 involving the abdominal cavity).

From 2018–2019 after partial implementation of the colorectal bundle actions 126 were included and 15 infections occurred (2 superficial, 4 deep and 9 involving the abdominal cavity). Concerning the three time periods, data acquisition is retrospective for the first two periods and prospective for the final period.

### Inclusion and exclusion criteria

All patients aged 18 years or older undergoing elective or emergency colorectal surgery from January 2021 until December 2021 will be included in the study. Written informed consent will be obtained. Patients under 18 years of age, pregnant patients or patients who refuse or are unable to give informed consent due to mental status, will be excluded from the study.

### Implementation of the colorectal bundle

In our study protocol we defined three time periods in order to evaluate the effectiveness of our colorectal bundle. We collected data and analyzed the SSI rate from October 2018 till September 2019, before our bundle was implemented. From October 2019 till September 2020, a first set of measures was established. Therefore, this time period is defined as “during implementation”. In January 2021 the full extent of our colorectal bundle was implemented and the time period “after implementation” started for one year.

Following measures were defined based on a current literature review.

1. There are multiple studies assessing the different methods of hair removal prior to surgery [8], such as shaving, clipping and chemical depilation. Compared with shaving, clipping leads to a significant reduction of SSI [9]. During shaving, the skin experiences microscopic cuts which can lead to colonization of microorganisms and thereby increases susceptibility for post-operative wound infections. Therefore, our first action is to remove hair with a clipper.
2. Surgery leads to a release of catabolic hormones as well as decrease in pancreatic beta-cell function, causing stress-induced hyperglycemia. Several studies have shown the association between hyperglycemia and elevated risk for SSI. Close glucose monitoring leads to a reduction of SSI, especially in non-diabetic patients [10]. A risk of hypoglycemia is described, but no serious adverse events occurred during treatment [11]. The second action is therefore the monitoring of blood glucose and the treatment with either insulin or glucose according to our scheme.
3. The application of antibiotics in order to reduce SSI is well established [12]. Studies show that the optimal time window for surgical antimicrobial prophylaxis is 30–59 minutes prior to incision [13]. Accordingly, our third action is to apply an antibiotic prophylaxis within the time frame.
4. Zanetti et al. could demonstrate that the redosing of prophylactic antibiotics during cardiac surgery can significantly reduce SSI [14]. Subsequently, the fourth action is to repeat the intravenous antibiotics if the operation lasts more than four hours.
5. The fifth action focuses on optimal body temperature. Perioperative hypothermia leads to SSI [15,16]. The application of warming systems reduces infections in colorectal surgery [17]. If core temperature is below 35°C, additional warming tools are applied, such as warm intravenous fluid. There is limited data on adverse effects, such as thermal burns.
6. The sixth action includes glove and instrument changes after finishing the anastomosis. Wearing the same gloves over a long period of time may cause microbiological contamination. Hence, in arthroplasty surgery, changing gloves was shown to significantly reduce SSI [18]. A change of instruments prevents a potential bacterial contamination of the abdominal cavity.
7. The seventh action is the use of wound protectors. In laparoscopic as well as in open surgery, dual ring wound protectors lead to a reduction of SSIs [19]. Most likely, a barrier between surgical field and wound can decrease bacterial contamination [20]. Prevention of temperature loss of the wound leads to an improved blood perfusion and reduces SSI.
8. The eight action concerns the surgeons’ experience. Colorectal operations are exclusively performed with the support of a well experienced consultant surgeon.
9. Our ninth and last action focuses on preoperative showering. Torres et al. have shown that a shower, even with customary soap the night before surgery, can significantly reduce SSIs [21].

As previously mentioned, our colorectal bundle was implemented gradually over a time period of three years. In the following, a detailed implementation plan is listed.

#### Pre-implementation phase (October 2018–September 2019)

During this time period, 137 patients underwent colorectal surgery. Only basic measures for preventing SSI were performed:

1. Hair was removed in the operating field with a clipper instead of a shaver.
2. Blood glucose was monitored only during the operation.
3. Antibiotic prophylaxis was applied 60 minutes before the operation.
4. The application of an antibiotic prophylaxis was repeated if the operation lasted longer than 4 hours.
5. Measures of warming were only applied during the operation and in the recovery room.
6. Instruments and gloves were changed after finishing the anastomosis.

#### Implementation phase (October 2019–September 2020)

During this phase, additional measures have been introduced:

1. Implementation of wound protectors during colorectal surgery.
2. Colorectal operations were only performed with the support of an experienced consultant surgeon.

#### Post-implementation phase (January 2021 – December 2021)

During this phase, additional measures are being implemented.

1. Close monitoring of blood glucose. During the stay in the recovery room and for 48 hours post-operative, blood glucose is monitored closely. Moreover, if blood glucose is higher than 9 mmol/l, the patient will be treated with insulin. This measure is applied to diabetic as well as to non-diabetic-patients.
2. The measures of warming are intensified. First of all, during the operation and during the stay in the recovery room, patients will be placed on warming mattresses. Moreover, a warming towel will be placed on the operating field directly after the operation.
3. The patients will be asked to take a shower the night before surgery. In case of an emergency operation, patients will be asked to wash the axilla, trunk, genitalia, groins and umbilicus.

The primary endpoint of this study is occurrence of SSI within 30 days after surgery. Evaluation of risk factors for SSI will be secondary endpoints. In ***Table 1***, the patient-associated risk factors and procedure-associated risk factors assessed in our study protocol are listed.

**Table 1:** Questionnaire for SSI Risk factors.


PATIENT ASSOCIATED RISK FACTORS	PROCEDURE ASSOCIATED RISK FACTORS

Age	Date of the operation?

Sex	Duration auf the operation (min)

Diagnosis	What kind of operation was performed?

Weight	Which technique of surgery was done (open, laparoscopic, robotic)?

Fat tissue measured in CT scan	Which type of stoma was inserted?

Height	

BMI	

Diabetes and what kind of diabetes	

HbA1c	

Alcoholism	

Immune-deficiency diseases and which type of disease exactly	

Smoking	

Immunosupressive drugs and which ones	

Chemotherapy	

Radiotherapy	

COPD	

pAVK	

Renal insufficience	

Length of hospitalization	

Pre-Albumin	

ASA Score	

Contamination Class of wounds	


Moreover, we measure pre-albumin levels to determine the nutritional status. We assess whether immune-modulatory supplements like Oral Impact^®^ have been given before surgery. We include the ASA (American Society of Anesthesiologists) score in our data to define the general health status of the patient. Furthermore, we classify the grade of contamination of the wound prior to surgery.

After the operation, we assess compliance by evaluating whether the nine measures of our colorectal bundle of care were performed as planned. If SSIs occur, we classify them and treat them according to the guidelines. Patients will be followed for the occurrence of SSIs within 30 days after surgery by means of a telephone survey. These telephone surveys have also been done during the pre-implementation and implementation phase. Our goal-directed questions enable patients to recognize symptoms suspicious of a SSI at home. Criteria for wound infection comprise redness and pain around the wound, drainage from cloudy fluid, fever, wound dehiscence, etc. If patients report suspicion of SSI, we strongly advise them to present themselves at our outpatient clinic or at the family doctor for evaluation and treatment.

### Statistical methods

Data will be presented as absolute and relative frequencies for categorical variables and using descriptive statistics for continuous variables.

The primary endpoint, occurrence of SSI within 30 days after surgery, will be determined as a rate (number of patients experiencing SSI/number of patients undergoing colorectal surgery). The SSI rate will be determined together with a 95% confidence interval according to Clopper-Pearson.

The SSI rate will be compared to the SSI rates obtained for previous years (pre-implementation phase implementation phase) in an unadjusted manner as well as after adjustment for NNIS risk index. The NNIS risk index will be applied according to the methodology of Swissnoso and is based on ASA score, surgical wound classification and duration of surgery.

Potential risk factors of SSIs will be evaluated in an exploratory manner by measures of association and utilizing logistic regression models.

The sample size in this observational study is based on the expected number of patients undergoing colorectal surgery annually. With approx. 150 patients, the half-width of the 95% confidence interval for the SSI rate will not exceed 6.5% as long as the SSI rate is not greater than 18%.

### Ethical approval

The Swiss Ethics Board has approved the trial’s protocol version 2, dated 21 April 2020. The trial will be performed according to the ethical protocol and the current version of the World Medical Association Declaration of Helsinki and the International Conference on Harmonization Gool Clinical Practice (ICH GCP) guidelines.

## Discussion

SSIs are a major challenge in patient care after colorectal surgery. A high number of SSIs leads to an increase in hospital length of stay, readmission rates, and healthcare costs [3]. Over the last years, several studies have shown that the combination of multiple SSI reduction measures into a bundle can significantly reduce SSI rates [5]. The impact of the individual measure and thus the “right” combination of a bundle of care for a single institution remains unclear [2].

The rate of SSIs is considered as quality factor for surgical care and thus we aimed to improve outcome quality with the implementation of a bundle of care in colorectal surgery at our institution. Since a variety of risk factors contribute to SSIs, it is widely believed that improvement cannot be achieved by one single action, even though each single action has been shown to reduce SSIs significantly in former studies. As a consequence, the implementation of a bundle of measures is necessary. Nevertheless the effectiveness and moreover the achievability of compliance and adherence to all these measures by a multidisciplinary team, is still under discussion. On the one hand, the positive effect of reduction of SSIs has been demonstrated in former studies [3]. On the other hand, some studies have shown that although every single action has a high level of evidence the bundle of care itself cannot reduce the rate of SSIs [22,23,24].

In our institution, an increasing number of SSIs in patients undergoing colorectal surgery could be observed over the last years. To improve patients` outcomes a colorectal bundle of care was gradually implemented over three years as a monocentric, prospective observational study.

To address and prevent malcompliance in a large multidisciplinary team repetitive instructions and trainings have been performed and for each action and team respectively a separate checklist was created. In addition, we plan meetings with the responsible team members on a monthly basis to update and raise awareness.

Several bundles with numerous separate actions have been described in literature. We have reflected our workflows and selected a comparatively high number of nine measures, which should be most effective in our setting to decrease the rate off SSIs. Regular information and instruction of all the involved teams and the rising of awareness towards this topic, can lead to behavioral changes contributing to a decrease of SSIs.

Our study has some limitations that have to be acknowledged. First of all this is a single-center study. We assume that the bundle will lead to a decrease of SSIs in our institution, but whether our measures can be applied successfully in other institutions, is difficult to predict. Secondly, the value of each individual measure is difficult or impossible to evaluate and this poses a problem in the interpretation of the effects of a whole bundle of care. Thirdly, our study has three periods of implementation of the measures of the colorectal bundle; while two periods were analyzed retrospectively, data of the main study period is acquired prospectively. Lastly, since different measures and combinations of measures are used in different bundles, comparability in literature is limited.

Besides analyzing the effects of our colorectal bundle on SSIs, we aim to investigate the impact of risk factors like smoking habits, alcoholism, weight, etc. and their combination.

This investigation is part of a quality improvement project to reduce SSIs and their consequences for the patients and the whole healthcare system.

Originally the timelines were from October 2020 to September 2021, but due to the Corona pandemic we had to alter the timeline from January 2021 to December 2021.

## Data Accessibility Statement

The datasets used and/or analyzed during the current study are available from the corresponding author on reasonable request.
